# Cellular and molecular mediators of lymphangiogenesis in inflammatory bowel disease

**DOI:** 10.1186/s12967-021-02922-2

**Published:** 2021-06-10

**Authors:** Dickson Kofi Wiredu Ocansey, Bing Pei, Xinwei Xu, Lu Zhang, Chinasa Valerie Olovo, Fei Mao

**Affiliations:** 1 Key Laboratory of Medical Science and Laboratory Medicine of Jiangsu Province, School of Medicine, Jiangsu University, 301 Xuefu Road, Zhenjiang, 212013 Jiangsu People’s Republic of China; 2 Directorate of University Health Services, University of Cape Coast, Cape Coast, Ghana; 3 Department of Clinical Laboratory, The Affiliated Suqian First People’s Hospital of Nanjing Medical University, Suqian, 223800 Jiangsu People’s Republic of China; 4 Department of Microbiology, University of Nigeria, Nsukka, 410001 Nigeria

**Keywords:** Inflammatory bowel disease, Lymphangiogenesis, Lymphatic system, Immune cells, Molecular pathways, Gut microbiota

## Abstract

**Background:**

Recent studies reporting the intricate crosstalk between cellular and molecular mediators and the lymphatic endothelium in the development of inflammatory bowel diseases (IBD) suggest altered inflammatory cell drainage and lymphatic vasculature, implicating the lymphatic system as a player in the occurrence, development, and recurrence of intestinal diseases. This article aims to review recent data on the modulatory functions of cellular and molecular components of the IBD microenvironment on the lymphatic system, particularly lymphangiogenesis. It serves as a promising therapeutic target for IBD management and treatment. The interaction with gut microbiota is also explored.

**Main text:**

Evidence shows that cells of the innate and adaptive immune system and certain non-immune cells participate in the complex processes of inflammatory-induced lymphangiogenesis through the secretion of a wide spectrum of molecular factors, which vary greatly among the various cells. Lymphangiogenesis enhances lymphatic fluid drainage, hence reduced infiltration of immunomodulatory cells and associated-inflammatory cytokines. Interestingly, some of the cellular mediators, including mast cells, neutrophils, basophils, monocytes, and lymphatic endothelial cells (LECs), are a source of lymphangiogenic molecules, and a target as they express specific receptors for lymphangiogenic factors.

**Conclusion:**

The effective target of lymphangiogenesis is expected to provide novel therapeutic interventions for intestinal inflammatory conditions, including IBD, through both immune and non-immune cells and based on cellular and molecular mechanisms of lymphangiogenesis that facilitate inflammation resolution.

## Background

IBD, including Crohn’s disease (CD) and ulcerative colitis (UC), is characterized by a dysregulated immune response to normal gut microbial antigens and genetically affects predisposed individuals. CD is an intestinal transmural inflammatory disease in which reactive changes are observed in mesenteric lymph nodes. Evidence suggests that reactive lymphadenopathy is a recognized cardinal sign of CD [1]. It was suggested in early 1946 that a CD is more common in the intestinal tract where lymphatic follicles are concentrated. The evidence of lymphatic alteration being an important factor in the pathogenesis of IBD is stronger in CD than UC. A growing body of evidence suggests that IBD is often accompanied by intestinal lymphatic aberrations, inflammatory lymphatic proliferation, and lymphangiogenesis [2, 3]. However, whether lymphangiogenesis represents a component of the pathology or a salutary attempt to resolve the inflammation is still an open question. Several scholars have indicated that lymphangiogenesis facilitates clearance of inflammatory cells, cytokines, and antigens from the inflamed site and promotes resolution of inflammation [4, 5]. Therefore, it is enticing to agree to these findings that lymphangiogenesis plays a key role in the modulation of inflammation in IBD.

Lymph vessel density increases in the intestinal mucosa of patients with IBD. Studies reporting abnormalities such as lymphangitis, immune cell trafficking, and lymphangiogenesis in IBD patients suggest impaired lymphatic drainage and lymphatic pumping, implicating the lymphatic system as a noticeable player in IBD pathogenesis [6, 7]. It is noteworthy that local tissue inflammation leads to a transient and profound remodeling of draining lymph nodes (DLNs), with volume dilation, lymphoid hyperplasia, and a marked increase in lymphatic vessel density in IBD [8, 9]. The increase in lymphatics may be an adaptation against tissue edema and accumulation of immune cells. A study that revealed the clinical monitoring significance of lymphatic vessel density in IBD suggested that decreased lymphatic vessel density is associated with postoperative endoscopic recurrence in CD [10]. Compared with blood vessels that regress rapidly after the resolution of inflammation, the new lymphatic vessels can persist once formed [11]. Baluk and colleagues report that despite the decreased leukocyte flow and the diminished stimulation of lymphangiogenesis following the amelioration of inflammation, lymphatic vessels formed during infection persist, ready for the next infection [12]. The significance of this lymphatic vessel persistence is unclear; it may reflect measures to prepare the immune system for subsequent events of insult or for lymphatic vessels to serve as immunological brakes to suppress subsequent immune responses [13]. The specific mechanisms protecting lymphatic vessels from regression remain to be elucidated. Again, persistent lymphatic vessels' main mediators and functions post recovery from intestinal inflammation or antibiotic treatment are yet to be defined.

While an increase in the other forms of lymphatic alterations, including lymphadenopathy, lymphangiectasia, lymphangitis, and lymphatic vascular occlusion, denotes poor prognosis, an increase in lymphangiogenesis indicates a good prognosis. Studies have therefore focused on investigating the factors that modulate lymphagiogenesis as a means to understanding the pathogenesis of IBD and exploring therapeutic targets [14–16]. Within the IBD microenvironment, several cells and molecules directly or indirectly influence and modulate the lymphatic system via upregulating or downregulating lymphangiogenesis. Adequate exploration of these cellular and molecule mediators is necessary to provide treatment targets for IBD. This paper examines available literature on the contribution of cellular mediators and molecular pathways associated with lymphangiogenesis in IBD and the interaction with gut microbiota, as these mediators serve as novel therapeutic targets in IBD management and treatment.

## Cellular mediators of lymphangiogenesis in IBD

The lymphatic system helps maintain tissue homeostasis, including tissue fluid balance, interstitial protein transport, and the development of cellular immunity. It contains a large number of cells such as macrophages, lymphocytes, and antigen-presenting cells that together trigger a primary immune response, in addition to specialized LECs in the cortex that contribute to the primary immune response via recruiting intravascular lymphocytes. In IBD-associated lymphatic changes, there is mixed inflammatory cell response with significant increases in T-helper (Th), T-regulatory (Treg), neutrophils, macrophages, and dendritic cells (DCs), among other immune cell populations. These cellular components directly or indirectly participate in regulating lymphangiogenesis in the inflammatory environment [17].

### Lymphatic endothelial cells

In the mammalian embryo, lymphatic endothelial progenitors bud from the cardinal vein and intersomitic vessels [18] with their identity and number maintained by the Prospero homeobox 1 (Prox1)-vascular endothelial growth factor receptor 3 (VEGFR3) feedback loop [19]. LEC progenitors produce Prox1, an important transcriptional factor for differentiation of LECs, in the cardinal vein of embryonic day (E) 10–11.5 mice. LEC progenitors accumulate to form pre-lymphatic clusters which sudsequently exit the cardinal vein wall to constitute superficial lymphatic vessels and lymph sacs [18, 20]. The VEGF-C induces the sprouting from the lymph sac through its receptor VEGFR, triggering receptor modulators such as β1 integrin and Ephrin B2 Neuropilin 2, to produce the lymphatic vascular network. A primary lymphatic network undergoes remodeling to become a mature lymphatic network composing of initial lymphatic vessels, collecting vessels, and pre-collectors [21–23]. LEC-specific ADP ribosylation factor 6 (Arf6) [21] and Pannexin-1 [24] also play pivotal roles in the formation of lymphatic vessel networks and the regulation of lymphangiogenesis. It is reported that knockdown of Arf6 in human LECs prevents in vitro directed cell migration and capillary tube formation triggered by VEGF-C via the inhibition of VEGF-C-induced internalization of β1 integrin [21]. Moreover, the loss of LEC-specific CPT1A (carnitine palmitoyltransferase 1A), a rate-controlling enzyme in fatty acid β-oxidation, inhibits lymphatic development. LECs employ fatty acid β-oxidation to facilitate their proliferation and epigenetic modulation of lymphatic markers expression during LEC differentiation. In this process, the transcription factor Prox1 triggers increased CPT1A expression, which upregulates the production of acetyl coenzyme A, dependent on fatty acid β-oxidation. The expressed acetyl coenzyme A is utilized by the histone acetyltransferase p300 to acetylate histones at lymphangiogenic genes, thus promoting lymphangiogenesis [25, 26].

Except for the central nervous system, bone marrow, and avascular tissues such as the cornea and epidermis, lymphatic capillaries are found in the skin and most internal organs [8]. In the resting state, LECs are normally quiescent [27]. In the course of inflammatory or malignant lesions, LECs can undergo proliferation or lymphangiogenesis under the stimulation of inflammatory or physicochemical factors. Adhesive interactions such as leukocyte rolling, adherence, and transendothelial migration regulated by various integrins, chemokines, and adhesion molecules between leukocyte and LECs are recognized to represent an early and rate-limiting step in the leukocyte infiltration. It is often accompanied by neoplastic tissue lesions associated with acute and chronic inflammatory diseases of the intestinal tract, ultimately resulting in a net up-regulation or down-regulation of the inflammatory response [28, 29]. However, extensive lymphangiogenesis, a sequence of processes including sprouting, migration, proliferation, and tubule formation by preexisting LECs, induced by leukocyte including macrophages, DCs, and neutrophils which generate diverse VEGF in response to the stimulus may play an important role in draining inflammatory macromolecules, immune cells, and debris from inflamed tissues [30, 31].

### Macrophages

During inflammation, macrophages are actively involved in inducing lymphangiogenesis [5, 13, 32, 33]. Meanwhile, LECs express chemotactic molecules that promote macrophage infiltration. The macrophages, in turn, secrete paracrine prolymphangiogenic growth factors such as VEGF-C, VEGF-D, and VEGF-A in response to inflammatory stimuli. One hypothesis has been proposed that lymphangiogenic macrophages can become incorporated into the lymphatic vessels and transdifferentiate into LECs, but it still requires more validation [34]. Increased levels of prolymphangiogenic growth factors (particularly enriched in CD11b+ macrophages from the draining lymph nodes [DLNs]) enhance the formation of new lymphatic vessels, which act to reinforce the recruitment of macrophages. Prolymphangiogenic growth factors secreted by infiltrating macrophages in inflamed tissue and DLNs appear to be critical in lymph flow, antigen clearance, and mobilization of inflammatory cells from inflamed tissue to the DLNs through enhancement of lymphangiogenesis and lymphatic vessel expansion [35, 36]. The recruitment of monocyte/macrophages and neutrophils via interleukin (IL)-1β in an inflammatory model induced lymphangiogenesis and up-regulated VEGF-C, VEGF-A, and VEGF-D. Conversely, the depletion of macrophages inhibited lymphangiogenesis and its associated factors [37]. Moreover, Zampell and colleagues demonstrated that CD4+ cells (macrophages, monocytes, Th cells, and DCs) regulate lymphangiogenesis during inflammatory lymphangiogenesis and wound repair in response to lymphatic fluid stasis [17].

The notion that lymphangiogenesis occurs mainly through paracrine action by a large number of infiltrating macrophages has been demonstrated, during which the role of toll-like receptor 4 (TLR4) in LECs supported the crucial role of macrophages in lymphangiogenesis via the lipopolysaccharide (LPS)-induced peritoneal inflammation model [38, 39]. It is reported that the inhibition of lymphangiogenesis is related to reduced lymphatic drainage and decreased inflammatory cell mobilization from the inflamed tissue to the DLNs in 2 different animal models of experimental IBD [5]. Moreover, macrophage mobilization plays an indispensable role in antigen clearance from the inflamed colon to the DLNs described in the same study [5]. The critical role of macrophages/monocytes in intestinal inflammation-associated lymphangiogenesis is further confirmed by Becker and colleagues. They proposed a dual role of the macrophage in promoting acute inflammation and contributing to inflammation-associated lymphangiogenesis [40]. It is possible to speculate that macrophage-mediated lymphangiogenesis in IBD may represent an attempt to compensate for the impaired lymphatic drainage to maintain a clearance of interstitial fluid and inflammatory infiltrate, thus restoring gut homeostasis. The observation that IL-10 suppresses VEGF from bone marrow-derived M1 macrophages but not VEGF production from M2 macrophages, and that activation signals determine the influence of IL-10 on VEGF production, further supports the idea that macrophages can adapt to their environment and respond with a coordinated set of signals to promote or resolve inflammation [41]. However, the role of macrophage polarization in lymphangiogenesis during the development of IBD remains to be explored.

### Neutrophils

Neutrophils are mostly considered pro-inflammatory cells since their involvement in inflamed sites in IBD, rheumatoid arthritis, and psoriasis correlates with the degree of tissue damage [42]. Available studies show that neutrophils contribute to regulating lymphangiogenesis in the inflamed environment. Neutrophils primarily regulate lymphangiogenesis by modulating VEGF-A bioactivity and bioavailability, and to a lesser extent, expressing VEGF-D [43]. In the inflammatpry-associated lymphangiogenic process, it is easy to assume that neutrophils cooperate with macrophages, considering the integral role played by macrophages in inflammation. Moreover, the same study documented that in the absence of B cells, intranodal lymphangiogenesis triggered in prolonged inflammation due to immunization, is associated with neutrophil accumulation, and that heparanase and matrix metalloproteinase 9 (MMP-9) obtained from neutrophils cooperate to upregulate VEGF-A bioactivity and bioavailability and in turn, inflammatory lymphangiogenesis [43]. However, this study was based on a model of skin inflammation, which is yet to be confirmed in gut inflammation. At the early stage of pregnancy and corpus luteum development in mammals, the major angiogenic factors, VEGF and fibroblast growth factor 2 (FGF2) contribute to the development of the corpus luteum. They may also act as a chemoattractant for polymorphonuclear neutrophils. Evidence indicates that these neutrophils are highest in the new corpus luteum where they interact with IL-8 to actively induce lymphangiogenesis and angiogenesis [44].

Impaired intestinal lymphatic drainage function in a mice model of experimental colitis, associated with elevated colonic neutrophil, macrophage, and T cell infiltration triggers lymphangiogenesis in addition to other functional (greater submucosal edema, higher immune cell burden) and structural (dilated tortuous lymphatic vessels) alterations in the intestinal lymphatic vasculature [45]. It is reported that although Ang-2−/− mice show the beneficial effect of reduced neutrophil infiltration and inflammatory angiogenesis in IBD, there is also blocked lymphatic maturation and expansion, resulting in dysregulated lymphatic function, which is detrimental in IBD. The inhibited lymphangiogenesis could be attributed to reduced migration of leukocytes especially neutrophils, into the inflamed gut since neutrophils infiltrate the inflamed gut in IBD provide several growth factors, cytokines, proteolytic enzymes, and oxidants which are significant contributors to tissue injury and inflammatory-associated lymphangiogenesis [46, 47].

### Dendritic cells

Based on phenotype, function, and development criteria, DCs are categorized into two main groups: plasmacytoid DCs (pDCs) and conventional or classical DCs (cDCs). The cDC produces VEGF-C via stimulation by IFN-γ from activated natural killer (NK) cells, when co-cultured with them. In turn, VEGF-C downregulates the expresion of interferon (IFN)-γ by NK cells. VEGF-C also encourages immune tolerance by enhancing the cross-presentation of self-proteins by MHC (major histocompatibility complex) class I in LECs. Thus, NK cell-dependent VEGF-C secretion might be part of a modulatory network involving DCs, NK cells, and LECs to maintain the needed balance between peripheral immune tolerance and inflammation [48, 49]. It is easier to speculate that cDCs contribute to lymphangiogenesis in secondary lymph nodes both indirectly and directly by secreting VEGF-C. In IBD, up-regulation of chemokines expressed by inflamed LECs and elevated interstitial fluid pressure may increase leukocyte flux from the intestinal mucosa into lymphatic vessels. The importance of this flow-induced mechanism is affirmed by studies that reported that chemokine (C–C motif) ligand 21 (CCL21) is barely produceed in the mouse tail lymphedema model, in which lymphatic flow was surgically blocked [50, 51]. In their study, Pflicke and Sixt indicated that immigration heavily relies on a C–C chemokine receptor type 7 (CCR7)-dependent mechanism, as CCR7-deficient DCs neither approached nor entered lymphatic vessels [52]. These observations suggest that CCL21 interaction with immune cells expressing its receptor, CCR7, provides the chemotactic gradient necessary to recruit leukocytes into lymph nodes, especially for DCs.

Regardless of DCs being linked with lymphangiogenesis in some experimental models of IBD, more direct evidence that they promote lymphangiogenesis is minimal. A study found that lymphadenopathy and lymphangiogenesis were still evident after intestinal inflammation recovery, and correlated with higher numbers of DCs in lymphatic and mucosal tissues [2]. The functional and morphological changes in the lymphatics could compromise DCs’ migration and function, potentially enhancing susceptibility to further intestinal disease and the recurrence of intestinal inflammation. The induction of DCs and macrophages with IgG immune complexes produce VEGF-A, which triggers VEGF-A-dependent intranodal lymphangiogenesis and increased DC level [53]. Moreover, DCs stimulate increased fibroblastic VEGF during the initial phase of vascular-stromal proliferation, implicating that lymph node-resident DCs orchestrate the initiation of lymphatic and blood vascular growth [54].

ALCAM (activated leukocyte cell adhesion molecule) is implicated in several pathophysiological processes, including leukocyte trafficking, lymphangiogenesis, and T cell activation. The inhibition of ALCAM downregulates antigen-presenting DCs activities and the transmigration of DCs across lymphatic endothelial monolayers, alongside reduced lymphangiogenesis in vivo and in vitro [55]. It is reported that lymphangiogenesis facilitates initial lymph formation, hence boosting the DCs' mobilisation of chemokine CCL21 [56]. These findings imply that lymphatic proliferation promotes initial lymph formation, thereby increasing the transport rate and reducing inflammatory mediators. Nevertheless, hyperplastic lymphatic vessels cannot induce increased immune cell migration despite an enhanced production of immune cell chemoattractants. The failure to collect accumulating filtered fluid, including immune cells and some types of antigens, aggravates lymphocytic lymphangitis in IBD. DC trafficking in tissues decreases in mice with defective dorsal meningeal lymphatic vessels but and increases in mice with enhanced dorsal meningeal lymphangiogenesis [57].

### Platelets

**Fig. 1:**
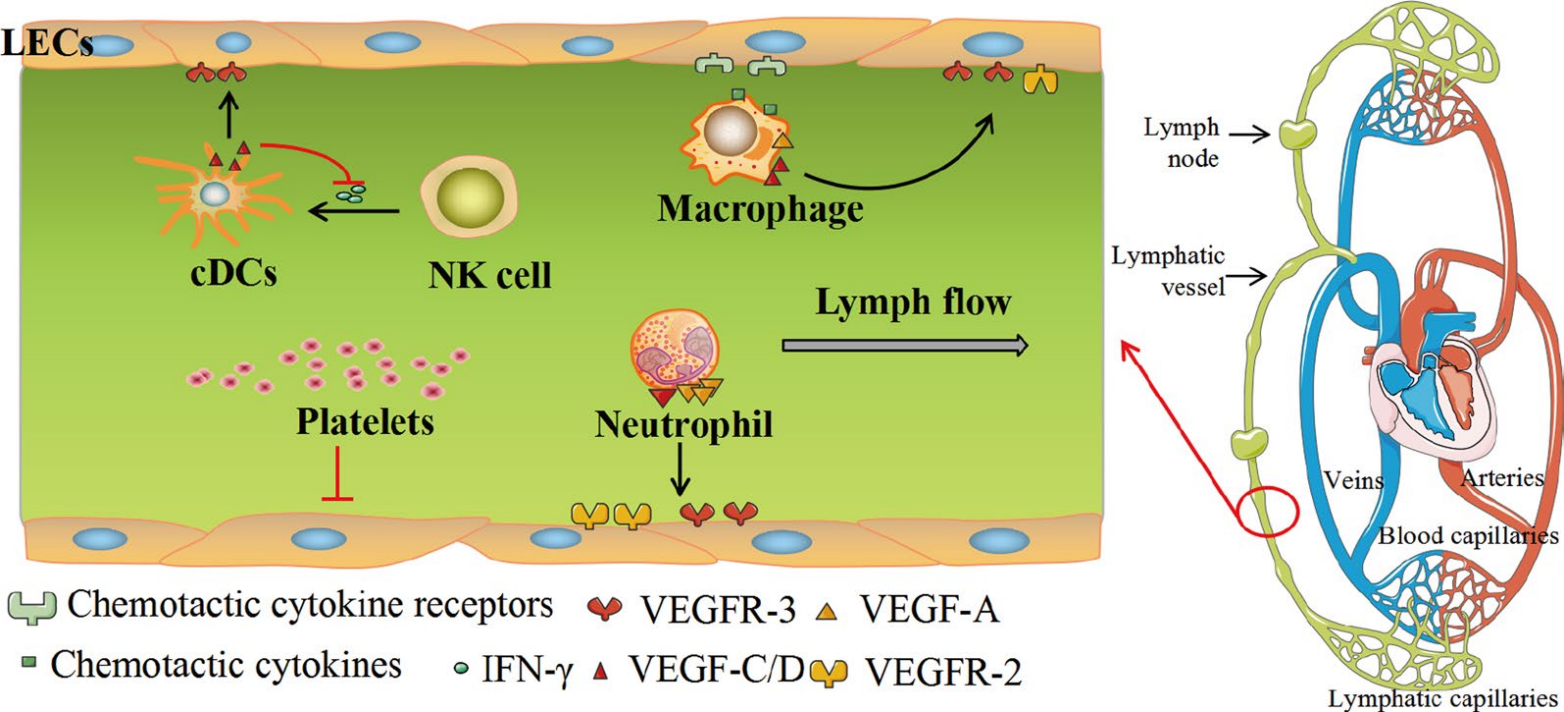
Cellular mediation of lymphangiogenesis. Both immune cells and non-immune cells play a role in the complex process of inflammatory-associated lymphangiogenesis in vitro and in vitro. LECs express chemotactic molecules that promote macrophage infiltration. In turn, macrophages secrete paracrine prolymphangiogenic growth factors such as VEGF-C, VEGF-D, and VEGF-A in response to inflammatory stimuli. Neutrophils conduce to lymphangiogenesis primarily by modulating VEGF-A bioavailability and bioactivity, and to a lesser extent, secreting VEGF-D. However, the suppressive mechanism of lymphangiogenesis by platelets might further contribute to persistent inflammation. cDCs produce VEGF-C when stimulated by IFN-γ released from NK cells during co-cultured with activated NK cells. In turn, VEGF-C reduces the secretion of IFN-γ from NK cells

In investigating the effect of platelets in lymphangiogenesis in colonic mucosal specimens from IBD patients, the authors found that regardless of the upregulated levels of lymphangiogenic factors during colonic inflammation, platelets inhibited the proliferation of LECs and increased in migration to LVs, causing a suppressed lymphangiogenesis which leads to the aggravation of colitis through the blockade of inflammatory cells clearance [58]. Other studies affirm that C-type lectin-like receptor-2 (CLEC-2) in platelets bind to podoplanin in LECs when lymphatic vessels separate from cardinal veins at the developmental stage, resulting in platelet activation and the release of platelet granule contents which potently suppresses the migration, proliferation, and tube formation of LECs to facilitate blood/lymphatic vessel separation [59–61]. Conversely, the co-culture of platelets with podoplanin-positive monocytes (PPMs) augments the secretion of lymphangiogenic cytokines including IL-1β via podoplanin/CLEC-2 axis, which capably enhances the migration, viability, and proliferation of LECs in vitro and significantly increases lymphatic neovascularization and facilitates wound healing in nude mice [62]. This calls for more investigations to clarify the mechanisms underlying the opposite effects of platelet in the induction of lymphangiogenesis. Figure 1 illustrates the participation of the cells discussed above in the expression of lymphangiogenic factors and other chemokines to enhance lymphatic flow.

### B cells

B cells contribute to the intestinal inflammatory milieu in IBD. CD27^−^IgD^−^ B cells are reduced in blood, while CD27^−^IgD^−^ B cells are increased in gut-associated lymphoid tissue in IBD [63]. Lymph node B lymphocytes orchestrate lymphangiogenesis, which enhances elevated DCs migration from the periphery into lymph nodes [64]. The B cell-dependent expansion of the lymphatic network reveals a unique relationship between B cells, lymphatic vessels, and migratory DCs in inflammatory conditions. As a prominent growth factor for both lymphangiogenesis and angiogenesis, VEGF-A is produced in inflamed tissues and draining lymph nodes. B cells are a source of this growth factor. This is confirmed in a study in which transgenic mice that express human VEGF-A specifically in B cells not only induced lymphangiogenesis in lymph nodes but also triggered the expansion of lymph nodes and the development of high endothelial venules [65]. Similarly, IgG immune complexes induced-VEGF-A production by B cells triggered VEGF-A-dependent intranodal lymphangiogenesis and increased DC number [53]. Several other studies have demonstrated the significance of VEGF-A in the induction of the growth of lymphatic endothelial cells and lymphangiogenesis in inflammation [66–70]. B cells interact with fibroblastic reticular cells to drive mesenteric lymph node lymphangiogenesis partly through VEGF-A and VEGF-C produced by the B cells [71]. The growth of the lymphatic vasculature occurs in two distinct phases of vascular-stromal growth; an initiation phase characterized by increased vascular-stromal proliferation, and a subsequent expansion phase. While the initiation phase is CD11c(+) cell-dependent and T/B cell-independent, the expansion phase is rather B and T cells dependent [54].

### T cells

Elevated colonic levels of infiltrating cells in IBD, including T cells, are associated with structural and functional changes in the intestinal lymphatic vasculature [45]. The depletion of CD4+ cells in the inflammatory site attenuates lymphangiogenesis and the expression of VEGF-C and VEGF-D. Mechanistically, the RAMP1 (receptor activity modifying protein 1) signaling in immune cells, including T cells plays an important role in inflammation-associated lymphangiogenesis by increasing VEGF-C and VEGF-D expression [32]. It is reported that CD4(+) T cells interact with macrophages to enhance lymphangiogenesis and that both lymphangiogenesis and edema are significantly decreased in CD4(+) T-cell-deficient mice or lymphocyte-deficient Rag2(?/?) mice and macrophage-depleted mice. The underlying mechanism indicates that T helper type 1 (Th1) and Th17 cells induce macrophages to secrete VEGF-C, promoting lymphangiogenesis [72]. Lymphedema in the mouse-tail causes a mixed inflammatory cell response with significant upregulation in Th cells, Treg, neutrophils, DCs, and macrophages. Contrary to the observation presented above [32, 72], the depletion of CD4+ cells but not CD8+ or CD25+ cells greatly enhances lymphangiogenesis in both tail model lymphedema and an inflammatory lymphangiogenesis model [17].

### Monocytes

There are three main distinct subpopulations of human monocyte defined as the classical monocytes (CD14+ CD16+), intermediate (CD14+ CD16+), and non-classical monocytes (CD14− CD16+) [73]. Several inflammatory stimuli can activate VEGF production from human monocytes [74, 75]. In an inflammatory environment, monocytes can be easily induced to present lymphatic phenotypes by expressing specific lymphatic endothelial markers such as LYVE-1 (lymphatic vessel endothelial hyaluronan receptor-1), Prox-1, and Podoplanin [76]. However, in acute colitis, monocyte-depleted mice are protected from intestinal injury and exhibit decreased inflammation-associated lymphangiogenesis, which is reversed after the administration of wild-type monocytes to CCR2 mice. Although CCR2 deficiency does not attenuate inflammation in chronic colitis, it rather reduces inflammation-associated lymphangiogenesis [40]. It could be speculated that intestinal inflammation and inflammation-associated lymphangiogenesis occur independently since inflammation-associated lymphangiogenesis is decreased in the absence of monocytes/macrophages. Monocytes expressing TIE2 receptors are both lymphangiogenic [77] and angiogenic [78, 79], and podoplanin-positive monocytes are also involved in the enhancement of lymphangiogenesis [62]. Monocyte-derived Wnt5a is directly involved in the pathological response of inflammatory lymphangiogenesis. Moreover, Wnt5a acts via the monocyte-derived cells to modulate VEGF-C production and macrophage phenotype [80]. Thrombospondin-1 acts as an endogenous inhibitor of lymphangiogenesis by ligating CD36 on monocytic cells and also suppressing macrophage-expressed lymphangiogenic factors VEGF-C and -D [81].

### Mast cells

Several other effector cells of inflammation, including mast cells, basophils, and eosinophils are important sources of a wide spectrum of lymphangiogenic and angiogenic molecules. For instance, human primary mast cells express lymphangiogenic factors VEGF-C and VEGF-D, and angiogenic factors VEGF-A and VEGF-B, in addition to the receptors VEGFR-1 and VEGFR-2 [82], which regulate the development of lymphangiogenesis in IBD. Moreover, Sammarco and colleagues report that gastric mast cells release lymphangiogenic and angiogenic factors such as VEGF-C, VEGF-A, VEGF-F, C-X-C motif chemokine ligand 8 (CXCL8), and matrix metallopeptidase 9 (MMP9) [83].

### Basophils

Basophils obtained from peripheral blood of healthy individuals constitutively express mRNA for VEGF-A and VEGF-B [84]. Similarly, immunologically activated human basophils selectively express VEGF-A, VEGF-B, and Angiopoietin1, which further activates Tie2 on mast cells [82]. In IBD patients Eotaxin, a potent and selective chemoattractant for basophils and eosinophils is significantly upregulated in the serum of both active CD and UC patients, suggesting that this cytokine may play an important role in the pathogenesis of IBD [85]. Although basophils are key contributors of inflammation [86, 87], participate in tumor growth and metastasis [88, 89], and have been demonstrated in draining lymph nodes of cancer patients [90], the production of lymphangiogenic factors by these cells needs to be further evaluated.

### Eosinophils

An eosinophil is a potent inflammatory cell thought to play an important role in the pathogenesis of IBD [91]. Clinical studies in IBD patients have provided increasing evidence that eosinophils contribute to chronic intestinal inflammation with a clear indication of changes in cytokine, chemokine, and receptor mediator profiling [92, 93]. Inactive sites of IBD patient biopsies, copious numbers of eosinophils, neutrophils, and mast cells have been quantified [94, 95]. Increased lymphangiogenesis in fibroinflammatory areas of Riedel thyroiditis also contained eosinophils in addition to lymphocytes, and IgG4+ plasma cells [96]. Eosinophils also regulate the resolution of inflammation and draining lymph node hypertrophy [97], participate in lymph node trafficking and antigen presentation [98], and contribute to inflammation in lymph nodes [99]. Regardless, no study has currently implicated eosinophils in the production of lymphangiogenic factors, hence more studies are needed to further address this observation.

### Others cells

**Fig. 2:**
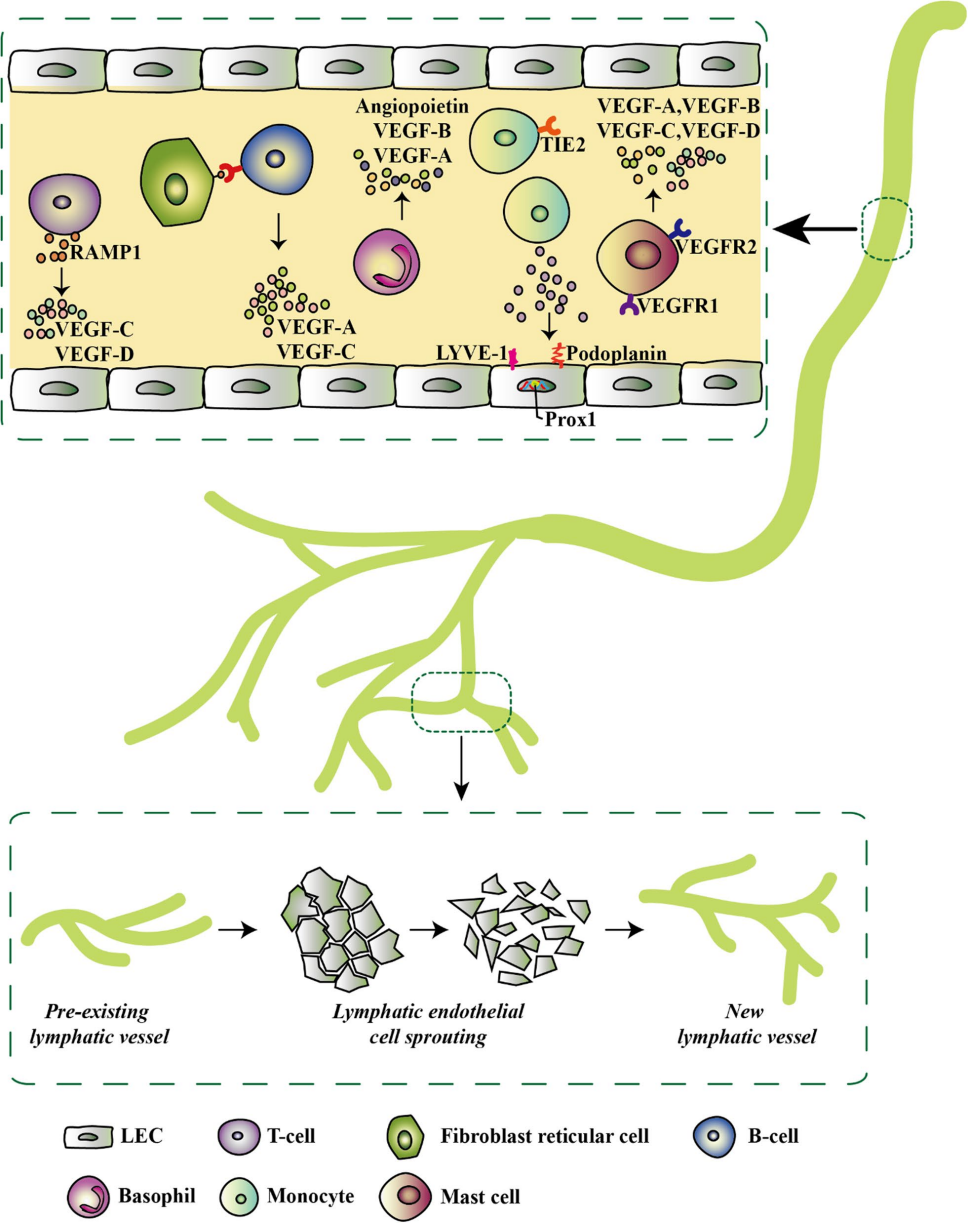
Inflammatory-associated cells and their secretome that initiate lymphatic expansion. Most of the inflammatory cells do not only secrete lymphangiogenic factors but also exhibit lymphangiogenic phenotypes by expressing specific lymphatic endothelial markers such as LYVE-1 (lymphatic vessel endothelial hyaluronan receptor-1), Prox-1, and Podoplanin. These factors trigger pre-existing lymphatic vessels in the inflammatory environment to give rise to new vessels via lymphatic endothelial cell sprouting

VEGF-C and -D are strong inducers of lymphangiogenesis and have essential (VEGF-C) and modulatory (VEGF-D) roles during developmental lymphangiogenesis. A myeloid population comprising largely of M2-polarized mononuclear cells and characterized by tyrosine kinase Syk's expression robustly expresses VEGF-C/-D, among other lymphangiogenic chemokines, and potently stimulate lymphangiogenesis in vivo [30]. The interactions between fibroblastic reticular cells and B cells promote mesenteric lymph node lymphangiogenesis. By regulating fibroblast reticular cells, the production of B-cell-activating factor can be promoted, and then the production of lymphangiogenic factors VEGF-A and VEGF-C is enhanced by B cells in collaboration with IL-4 [71]. Figure 2 summarizes the induction of lymphangiogenesis as discussed above.

## Molecular mediators of lymphangiogenesis in IBD

Several growth factors, cytokines, and chemokines participate in the regulation of lymphangiogenesis. These constitute coordinated molecular mechanisms and axes that directly or indirectly drive lymphatic expansion. The molecular mediators of lymphangiogenesis are primarily expressed by interacting with the inflammatory environment's cellular components. The mechanisms of lymphatic formation have been extensively studied in the neonatal stage but less in adults [100]. LYVE-1 is the first indicator of lymphatic endothelial commitment and LECs that express LYVE-1 can differentiate into lymphatic vessels via signaling pathways involving transcription factors such as Prox-1 and VEGF-C/VEGF-R3. In the adult colonic inflamed site, several factors such as tumor necrosis factor (TNF)-α, IL-1β, and IL-6 stimulate inflammatory cells such as macrophages to induce lymphangiogenesis by secreting VEGF-C/D [101–103] as illustrated in Figs. 1 and 2. Activated platelets also interact with podoplanin to cause blood vessel/lymphatic vessel divergence, leading to the extension of lymphatic vessels. CLEC-2, a platelet-activating receptor, and podoplanin (an endogenous ligand for CLEC-2) expressed on LECs interact in this process. The interaction activates platelets and induces platelet aggregation, which blocks lymphatic-venous connections and facilitates the separation of lymphatic and blood vascular systems via the inhibition of proliferation, migration, and tube formation of LECs [60]. The suppressive mechanism of lymphangiogenesis by platelets might be responsible for prolonged inflammation in IBD [58]; therefore, modulating the interaction between platelets and lymphatics could be a new therapeutic target for managing IBD. Furthermore, the effects of the interaction between platelets and other immune cells in lymphangiogenesis are also worthy of investigation.

### VEGF-C/VEGFR3 signaling pathway

Lymphangiogenesis is mainly mediated by the lymphatic VEGF-C and VEGF-D binding to VEGFR-3 [104]. Researchers have investigated the effect of a stimulated lymphatic function on the adaptive immune response via VEGF-C/VEGFR3 signaling on various factors, including intestinal inflammation, lymphatic drainage, bacterial antigen clearance, and macrophage activation during gut inflammation. It is demonstrated that VEGFR-3 blockade significantly affects lymphangiogenesis, reducing both area density and lymphatic vessel dimension while significantly increasing inflammatory edema formation and inhibiting disease resolution [5]. This is consistent with other fairly recent studies in which blockade of VEGFR-3 aggravated IBD and lymphatic vessel enlargement [105], degradation of VEGFR-3 inhibited lymphagiogenesis [106], and VEGF-C/VEGFR3-dependent autophagy and polarization triggered lymphangiogenesis [107]. The systemic delivery of VEGF-C provides significant protection against acute and chronic colitis. Additionally, the VEGFR-3 signaling promotes human intestinal LECs proliferation, migration, and organization in vitro [5]. These findings demonstrate that stimulation of functional lymphangiogenesis via VEGFR-3 accelerates disease resolution and inhibits chronic inflammation in the experimental model of IBD.

Several immune cells, including macrophages, DCs, and mast cells, express VEGF-C [108]. For instance, the polarizing and resolving effects of macrophages are mediated by the VEGF-C/VEGFR3 pathway through signal transducer and activator of transcription (STAT)6 activation [5]. This is intriguing because some reports suggest that there may be a dysregulation of STAT6 signaling in the ungoverned immune response associated with colitis, wherein the activation of this transcription factor promotes mucosal repair and resolution of IBD [109]. However, there are conflicting findings that demonstrate that the stimulation of lymphangiogenesis by VEGF-C during acute colitis promotes inflammatory lymphangiogenesis in the colon and aggravates intestinal inflammation [42]. This implies that inflammatory lymphangiogenesis may have pleiotropic effects at different stages of IBD and that VEGF-C exhibits a dual effect in inflammation, making it an important therapeutic target for IBD.

### NF-κB signaling pathway

The key factors that regulate inflammation-induced transcription are members of the nuclear factor-kappa B (NF-κB) family. Multiple intracellular signaling cascades rely on NF-κB to orchestrate the transcriptional control of various pro- and anti-inflammatory mediators shaping inflammatory responses and homeostatic processes like growth control apoptosis [110, 111]. The primary mediators of the inflammatory response are dimeric transcription factors that belong to the NF-κB family consisting of RelA (p65), NF-κB1 (p50), RelB, c-Rel, and NF-κB2 (p52). In unstimulated cells, NF-κB is sequestered in an inactive form in the cytoplasm bound to specific inhibitory proteins of the inhibitor (I)-B family. After stimulation through TLRs, proinflammatory cytokines or antigen receptors, I-κB is phosphorylated, ubiquitinated, and finally proteolytically degraded, leading to de-repression of NF-κB, which rapidly translocate to the nucleus and activates the transcription of several target genes [112]. It has also been thoroughly established that intestinal inflammation occurring in IBD is accompanied by an NF-κB driven overexpression of pro-inflammatory adhesion molecules and mediators, leading to disturbances in mucosal immunity [113, 114].

Besides the classic targets (chemokines and cytokines), it has also been described that inflammation-induced NF-κB directly upregulates two major transcripts involved in lymphangiogenesis, VEGFR-3, and its key transcription factor Prox1 (Prospero-related homeobox-1), resulting in a sustained inflammatory-induced lymphatic formation [115]. A study identified VEGFR-3 and Prox1 as downstream targets of the NF-κB pathway in inflammation-induced lymphangiogenesis. The induction of the NF-κB pathway by inflammatory stimuli activates Prox1, and both NF-κB and Prox1 activate the VEGFR-3 promoter, leading to increased receptor expression in LECs. This, in turn, enhances the responsiveness of pre-existing lymphatic endothelium to VEGFR-3 binding factors, VEGF-C and VEGF-D, ultimately resulting in robust lymphangiogenesis [116]. The NF-κB pathway presents an auspicious mechanistic connection between a variety of inflammatory stimuli and inflammation-induced lymphatic hyperplasia as well as lymphangiogenesis through a Prox-1 regulated increase of VEGFR-3 expression, amplifying cellular responses to local growth factors like VEGF-C/D [115, 116]. The enhancement of lymphangiogenesis and lymphatic vessel remodeling also occurs via NF-κB/VCAM-1 (vascular cell adhesion molecule 1) signaling pathway in human LECs [117], and the NF-κB/HIF-1α (hypoxia-inducible factor-1α) axis [118].

The upregulation of VEGFR-3 expression by inflammatory cytokines through NF-κB and Prox-1 may result in increased receptor availability, but a lower net availability of local VEGF-C/D might potentially contribute to "aberrant" IBD associated intestinal lymphatic characteristics [115, 116, 119, 120]. This might lead to a shift in IBD toward deficient VEGFR-3 signaling. These findings may indicate that an IBD-associated depression in lymphatic activation (possibly through NF-κB pathway suppression) could lead to insufficient lymphatic density, loss of lymphatic specification, and inappropriate patterning or remodeling, any of which would be anticipated to intensify the disease activity of IBD [8, 121]. Regardless, it is still uncertain whether and when a lymphatic vessel expansion in IBD is accompanied by impaired or improved lymphatic function. As discussed above, the role of NF-κB in IBD-related intestinal inflammation is by now clearly established. However, there are still gaps in knowledge regarding inflammatory induced lymphangiogenesis through Prox-1 and VEGFR-3 and their activation growth factors (VEGF-C, VEGF-D), thus remaining a new and promising area. These findings further suggest that identifying mechanisms that support and induce expression of lymphatics in the inflamed intestine may reveal novel therapeutic approaches for treating IBD.

### TLR4 signaling pathway

Lymphatic endothelial progenitors originate from plastic myeloid cells induced through TLR4 [122], which is also important in lipopolysaccharide (LPS)-induced inflammation. Studies show that LECs produce high amounts of TLR4 in the intracellular region. The TLR4 of LECs is the main mediator of NF-κB activation in LPS-induced VEGF-C-dependent lymphangiogenesis [36, 123]. LPS-TLR4 signaling in LECs cuases the production of various chemokines for chemotaxis of macrophage, which in turn infiltrate the lymphatic vessel to contribute to the induction of lymphangiogenesis by expressing lymphangiogenic growth factors. Because NF-κB is persistently active in LECs to maintain their phenotypic specification, TLRs may provide key links between pattern recognition, lymphatic structure, maturation, and function. TLRs likely regulate responses towards lymphatically filtered antigens or assist LEC responses to internalized antigens.

The importance of the balancing role of TLR4 in intestinal injury and repair is also reported by Yun-Jie and colleagues, who found that moderate activation of TLR4 signaling both promotes inflammation and repairs the intestinal epithelium in DSS-induced colitis and radiation damage [124]. TLR4-mediated signaling is important for the recruitment of immune cells to the site of inflammation, promoting reparative mechanisms, but can be described as a double-edged sword, as aberrant stimulation can induce chronic inflammation and mesenteric lymphatic alterations in a TLR4-PAMP (pathogen-associated molecular patterns)/DAMP (-associated molecular patterns) discriminative manner [125]. The observation that reduced lymphangiogenesis improves DSS-induced phenotype implies that the restoration of lymphatic function to a “normal” phenotype, significantly aids the repair of DSS-induced disease activity, which is achieved through TLR4 blockade with C34 [125]. This apparent contradiction of the general observation that increased lymphangiogenesis improves IBD implies that a balance is required in the therapeutic induction of lymphangiogenesis to arrive at a maximum benefit.

### Other signaling pathways

There are many other signaling pathways involved in the development of lymphangiogenesis in IBD and its associated CRC. In recent years, sphingosine-1-phosphate (S1P) has been recognized for its role in inflammation and cancer as a multipotent lipid mediator [126]. The level of S1P concentration in the blood and lymph is important for regulating lymphatic transport in the pathology of inflammation. Key chemokines like IL-6 and TNF-α in IBD are also associated with S1P signal activation. S1P binds to SIP receptor 1 (S1PR1) and amplifies chronic inflammation through the S1P-STAT3-S1PR1 amplification ring, thus exacerbating the disease. Besides, the S1P gene is highly expressed in many cancer cells, such as colon cancer cells, where a high concentration of S1P are released to act on lymphatic endothelial cells and immune cells in the tumor microenvironment, induce lymphatic generation and metastasis and promote the invasion and lymphatic metastasis of colorectal cancer [126, 127].

Lacteals are lymphatic capillaries of the small intestine and play crucial roles in the gut immune response and dietary fat absorption. According to Bernier-Latmani and colleagues, lacteals reside in a permanent regenerative and proliferative state unique from embryonic lymphangiogenesis or quiescent lymphatic vessels present in other tissues. Notch signaling and its ligand, delta-like 4 (DLL4), expressed in lacteals via the activation of VEGFR2 and VEGFR3 ensure the continuous regeneration process [128]. The role of certain exosomal components such as microRNAs (miRNA) in lymphangiogenesis has been explored. Stem cells treated with VEGF-C express a high concentration of miR-132 which is directly transferred from the stem cells to the LECs by the mediation of exosomes, inducing LECs proliferation, migration, and tube formation. The VEGF-C-dependent lymphangiogenesis occurs via miR-132 directly targeting Smad-7 and modulating transforming growth factor (TGF)-β/Smad signaling [129]. As potent regulators of inflammatory-associated lymphangiogenesis, various sources of stem cells and their secretory products are being explored as a possible treatment option for IBD [130, 131]. The various pathways linked with lymphangiogenesis are summarized in Table 1.

**Table 1:** Molecular pathways regulating lymphangiogenesis in inflammatory bowel disease

Molecule(s)	Functions in the IBD	Mechanism	References
CELC2	Inhibits LEC-mediated lymphangiogenesis to cause a sustained inflammatory response in the IBD process	Activates platelets by interacting with the LECs surface ligand Podoplanin, to inhibit LECs-mediated lymphangiogenesis	[60]
VEGF-C/VEGFR3	Prevents chronic inflammation and promotes disease regression	Promotes in vitro proliferation, migration, and tissue formation of human intestinal LECs, ultimately leading to functional lymphangiogenesis which can alleviate IBD	[5]
VEGF-A and VEGF-D	Promote lymphangiogenesis and decrease local inflammation	Neutrophils increase VEGF-A bioavailability and bioactivity via the secretion of MMP-9, heparinase, and to a lesser extent VEGF-D	[43]
NF-ΚB	Result in inflammatory-induced lymphatic formation	NF-κB and Prox1 synergistic control of VEGFR3 expression lead to increased receptor availability, resulting in reduced local VEGF-C/D net availability to cause VEGFR3 signal deficiency, blocked lymphoid activation in IBD, and exacerbating the disease	[116, 117]
TLR4	Blocking TLR4 can reduce the formation of inflammatory lymphatic vessels and improve the enteritis phenotype induced by DSS	Highly expressed in LECs; it is the main regulating medium for LPS to activate NF-KB	[125, 132]
TLR4- NF-κB/JNK pathways	Promotes human dermal lymphatic endothelial cells' (HDLECs') capacity of tube-like formation in vitro and accelerates lymphangiogenesis and lymph node metastasis in nude mice via LPS induction	LPS increases VEGF-C expression to promote cell motility and lymphangiogenesis through the TLR4- NF-κB/JNK signaling	[123]
S1P	The concentration grade of S1P in lymph and carcinoma tissues affects lymphatic transport and lymphangiogenesis of cancer cells, thereby promoting inflammation progression and CAC metastasis	Through the S1P-STAT3-S1PR1 amplification ring, the lymphatic transport in inflammation is affected, thereby promoting the accumulation of pro-inflammatory factors such as IL-6 and TNF-α to aggravate the disease	[126, 127]
Notch/DLL4 signaling	The genetic inactivation of Dll4 in lymphatic endothelial cells led to lacteal regression	The continuous regeneration and proliferation of lymphatic capillaries are mediated by Notch signaling and the expression of the Notch ligand delta-like 4 (DLL4) in lacteals which requires activation of VEGFR3 and VEGFR2	[128]
NRP-2	Promote tumor cell migration, invasion, and lymph node metastasis	The activation of the VEGF-C/D-NRP-2 axis stimulates lymphatic sprouting, facilitates the extension of LEC growth leading edge, and promotes the polarization of LECs. This axis is also the foundation of the sprouting and formation of a new network of lymphatic vessels	[133]
EMILIN-1/α9β1	The downregulation of EMILIN-1 leads to an inhibited lymphangiogenesis and a blockage in the dissipation of inflammation	EMILIN1 interacts with integrin α9 in the lymphatic vasculature to promote lymphatic valve formation and maintenance and exhibits a modulatory function in the proliferation by acting as a "guiding" molecule in the migration of LECs	[134, 135]

## Lymphangiogenesis and gut microbiota

The gut microbiota composition is influenced by different factors, including host genotype, diet, immunological status, and environment. Delayed or ineffective elimination of bacteria that penetrate the intestinal mucosal barrier can result in a prolonged innate immune responses and encourage the presentation of bacterial antigens to promote the induction of adaptive immune responses. Hence, while IBD could lead to an aberrant immune response against intestinal bacteria, ineffective or slow clearance of local bacteria could also be a key contributing event associated with the disease pathogenesis [136]. Past studies characterizing the colonic microbiome typically relied on stool samples, which do not accurately recapitulate the composition of bacteria at the mucosal surface [137]. The mesenteric lymph nodes (MLNs)' microbiome correlates closely with that found at nearby all mucosal sites. Commensal bacteria, therefore, play specific roles in the gut immune system and lymphagiogenesis, distinguishable from the effect they would have if recognized by the systemic immune system. Gut microbiota regulates lacteal or small intestine lymphatic capillaries structure, and integrity as germ-depleted mice suffer significant lacteal regression during adulthood and delayed lacteal maturation during the postnatal period [138].

**Fig. 3:**
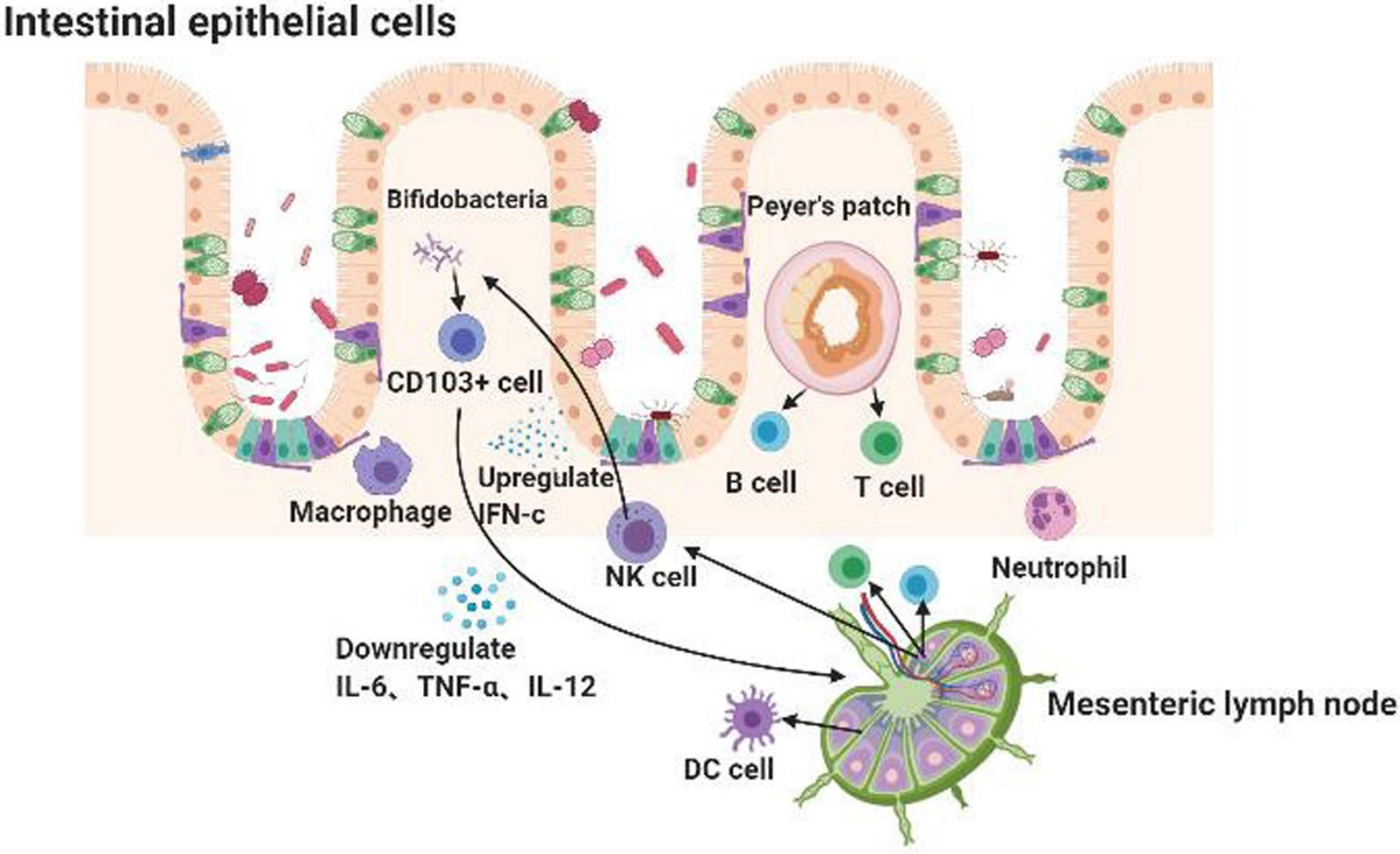
The interaction between the mesenteric lymphatics and the intestinal flora. Invading intestinal mucosal bacteria interact with various immune cells in mesenteric lymph nodes, such as T cells, B cells, DC cells, macrophages, NK cells, and neutrophils. When bacteria stimulate the mesenteric lymph nodes, more IFN-C is secreted. At the same time, bifidobacteria can lead to decreased IL-6, TNF-α, and IL-12 secretion of DC cells. Also, the presence of microorganisms can activate the CD40 pathway and make CD103+ cells migrate from lamina propria to MLN, playing an important role in immune regulation, hence lymphangiogenesis

Gut microbiota regulates the expression of both inflammatory and anti-inflammatory mediators that directly or indirectly contribute to the modulation of lymphangiogenesis. For instance, the production of IL-6, TNF-α, and IL-12 is downregulated by bifidobacteria induced in DC by *L. acidophilus*, which is also observed in splenic DC, but not in MLN DC. Meanwhile, MLN cells respond to bacterial stimulation with higher IFN-c production than spleen cells, possibly due to the presence of more responsive natural killer (NK) cells [139]. CD40-signalling as a microbe-independent signal, can trigger the migration of CD103+ DCs from the gut lumen for transport to draining lymph nodes and generate receptor-related orphan γt+ (RORγt+) Helios-induced Treg (iTreg) cells [140]. The participation of some gut bacteria in lymphatic modulation is illustrated in Fig. 3.

## Discussion and conclusions

A key function of lymphatics is to transport immune cells to the lymph nodes and drain interstitial fluid; thus, the blockade of lymphangiogenesis through any signaling means promotes the exacerbation of IBD and vice versa. Obstructed and dysfunctional lymphatics are known histopathological features of IBD. Although a dysfunction in the lymphatics might not be the direct consequence of the disease, the resultant inflammation presumably affects the lymphatic vascular function, aggravating an already compromised condition. The inflammatory modulators produced in UC and CD impair lymphatic fluid flow and change lymphatic vessel function, worsening tissue edema and accumulating bacteria and dead cells, which together exacerbate the existing inflammatory condition. Reports indicate that intestinal wall lymph edema of patients with IBD originates from obstructive lymphocytic lymphangitis. The inability of the lymphatic vasculature to collect accumulating filtered fluid, including the immune cells and some types of antigens, aggravates lymphocytic lymphangitis in IBD; therefore, enhancing lymphatic function is of key importance in the management of IBD.

Several cytokines, chemokines, and infiltrating and local cells induce lymphangiogenesis, which correlates with a good prognosis of IBD. For example, VEGF-induced lymphangiogenesis and increased tissue drainage inhibit acute and chronic inflammatory conditions such as IBD, wherein an activated lymphatic endothelium might mediate peripheral tolerance by establishing an immune-inhibitory microenvironment characterized by upregulated levels of Treg cells, immature CD11c+ CD11b+ DCs, and CD8+ cells exhibiting reduced effector function [140]. This implies that activated LECs induce lymphangiogenesis and potently suppress DCs maturation by reducing the expression of MHCII, CD40, and IL-6 while increasing IL-10 and CCL2 expression favoring an anti-inflammatory microenvironment. The exploration of the cellular and molecular mediators in this process is of great significance since it will help to clarify the exact molecular mechanisms of lymphatic changes in IBD, especially lymphangiogenesis to serve as a possible effective therapeutic target in IBD. Specific studies on the mesenteric lymphatic system in IBD have provided several effective and valid targets for therapeutic intervention and it is expected to provide novel therapeutic approaches to intestinal inflammation through both immune and non-immune cells, and based on cellular and molecular mechanisms of lymphangiogenesis that facilitate inflammation resolution.

## Data Availability

Not applicable.

## Abbreviations

ALCAM: Activated leukocyte cell adhesion molecule
CCL: C–C motif ligand
CCR: C–C chemokine receptor
CD: Crohn’s disease
cDCs: Conventional or classical DCs
CLEC-2: C-type lectin-like receptor-2
CPT1A: Carnitine palmitoyltransferase 1A
CXCL8: C–X–C motif chemokine ligand 8
DAMP: Damage-associated molecular patterns
DC: Dendritic cell
DLL4: Delta-like 4
DLNs: Draining lymph nodes
EMILIN-1: Elastin microfibril interface located protein
HIF-1α: Hypoxia inducible factor-1α
IBD: Inflammatory bowel diseases
IFN: Interferon
IL: Interleukin
LECs: Lymphatic endothelial cells
LPS: Lipopolysaccharide
LYVE-1: Lymphatic vessel endothelial hyaluronan receptor-1
MHC: Major histocompatibility complex
miRNA: MicroRNA
MLNs: Mesenteric lymph nodes
MMP9: Matrix metallopeptidase 9
NF-κB: Nuclear factor-kappa B
NK: Natural killer
PAMP: Pathogen-associated molecular patterns
pDCs: Plasmacytoid DCs
Prox1: Prospero homeobox 1
RAMP1: Receptor activity modifying protein 1
SIP: Sphingosine-1-phosphate
SIPR: Sphingosine-1-phosphate receptor
STAT3: Signal transducer and activator of transcription 3
TGF-β: Transforming growth factor
Th: T-helper
TLR: Toll-like receptor
TNF: Tumor necrosis factor
Treg: T-regulatory
UC: Ulcerative colitis
VCAM-1: Vascular cell adhesion molecule 1
VEGF: Vascular endothelial growth factor
VEGFR: Vascular endothelial growth factor receptor
α9β1: Integrin α9β1
